# Supplementary material for: Non-Targeted Metabolomics Approach Revealed Significant Changes in Metabolic Pathways in Patients with Chronic Traumatic Encephalopathy
Source: Biomedicines. 2022 Jul 15;10(7):1718. doi: 10.3390/biomedicines10071718 (PMC9313062; doi:10.3390/biomedicines10071718)
Supplement: Supplementary file 1 [file biomedicines-10-01718-s001.zip › biomedicines-1777223-supplementary.pdf]

Supplementary Information

Non-targeted Metabolomics Approach Revealed Significant Changes in Metabolic Pathways in Patients with Chronic Traumatic Encephalopathy

Jinkyung Lee, SuHyun Kim, Yoon Hwan Kim, Uiyeol Park, Junghee Lee, Ann C. McKee, Ky oung Heon Kim, Hoon Ryu, and Jeongae Lee

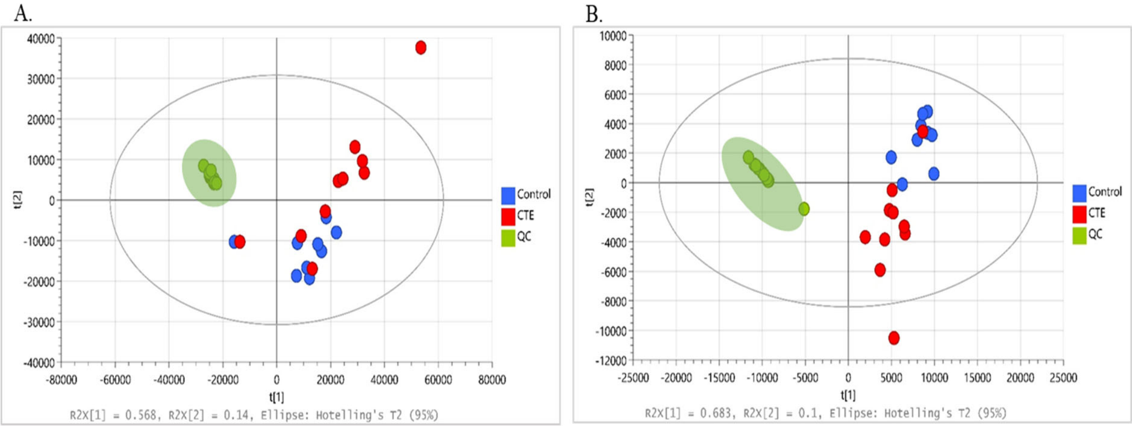

Figure S1. Principal component analysis (PCA) score plot of quality control (QC) samples. (**A**) In positive ion mode. (**B**) In negative ion mode. Clustering of QC samples (green) indicate that the raw data was acquired with a good repeatability.

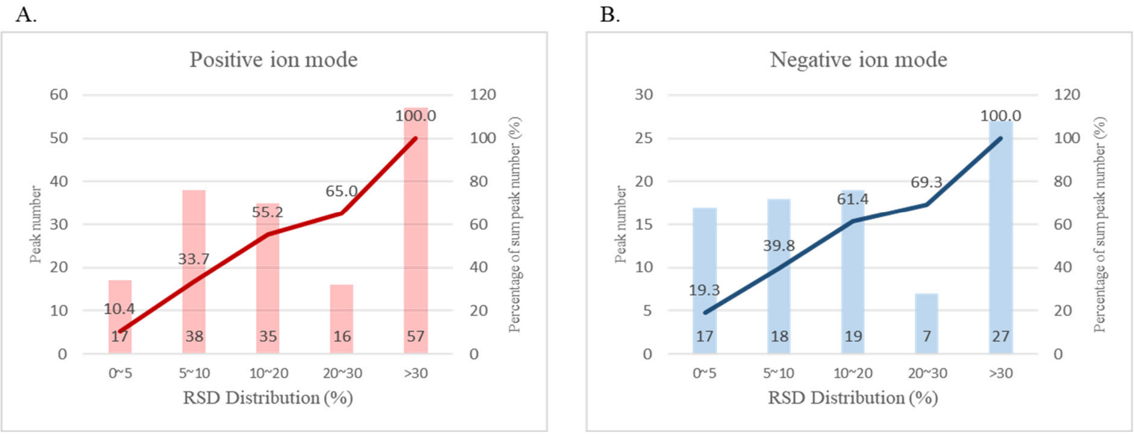

Figure S2. The relative standard deviation (RSD) of peaks in QC samples. (**A**) In positive ion mode. (**B**) In negative ion mode.

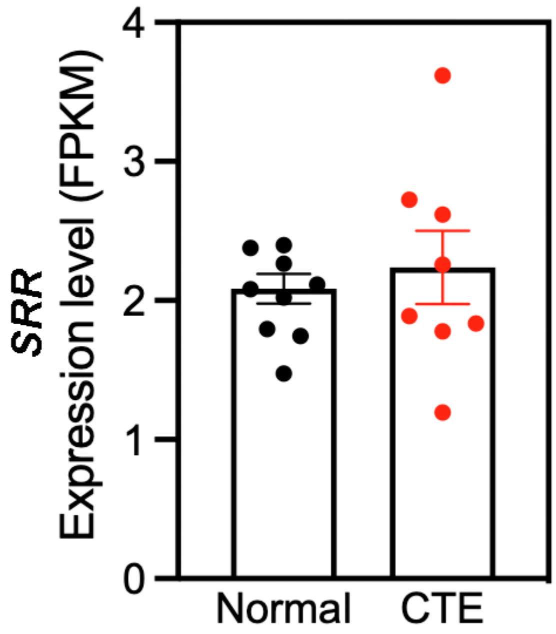

Figure S3. ***Serine racemase* (*SRR*) gene expression is slightly increased in CTE patients (*N* = 8) compared to normal subjects (*N* = 8).** *P* value is 0.136

Table S1. Information on the postmortem brain tissues (superior temporal cortex) from control subjects and CTE patients. CTE stages [from stages I (mild) to IV (severe)] were determined according to Dr. McKee criteria based on the density and regional deposition of hyperphosphorylated tau (p-tau) pathology. The criteria for pathological diagnosis of CTE were adopted and refined by the National Institute of Neurological Disorders and Stroke (NINDS)/National Institute of Biomedical Imaging and Bioengineering (NIBIB).

Number	Case	Sex	Age	CTE Stage
1	Control	Female	101	N/A
2	Control	Female	87	N/A
3	Control	Male	74	N/A
4	Control	Male	86	N/A
5	Control	Female	87	N/A
6	Control	Male	89	N/A
7	Control	Male	67	N/A
8	Control	Male	82	N/A
9	Control	Male	61	N/A
1	CTE	Male	82	IV
2	CTE	Male	66	IV
3	CTE	Male	58	III
4	CTE	Male	79	IV
5	CTE	Male	52	III
6	CTE	Male	69	III
7	CTE	Male	70	IV
8	CTE	Male	82	IV
9	CTE	Male	66	IV
10	CTE	Male	53	III

Table S2. Reversed-phase LTQ-Orbitrap-MS gradient elution program for ESI+ and ESI− modes.

Time (min)	Mobile phase A (%)	Mobile phase B (%)
0	100	0
3	100	0
10	50	50
12	10	90
12.5	100	0
14	100	0

Table S3. The detailed results of metabolic pathway analysis.

Pathway Name	Total	Hits	Raw p	−log(10)p	FDR	Impact
Tyrosine metabolism	42	4	0.0001	4.15	0.0014	0.2460
Arginine and proline metabolism	38	4	0.0017	2.76	0.0110	0.1169
Glycine, serine and threonine metabolism	33	4	0.0104	1.98	0.0237	0.2267
Aminoacyl-tRNA biosynthesis	48	4	0.0127	1.90	0.0237	0.1667
Phenylalanine, tyrosine and tryptophan biosynthesis	4	2	0.0151	1.82	0.0237	1.0000
Phenylalanine metabolism	10	2	0.0151	1.82	0.0237	0.3571
Nicotinate and nicotinamide metabolism	15	1	0.0162	1.79	0.0237	0.1943
Retinol metabolism	17	1	0.0321	1.49	0.0359	0.2165
